# Effects of Prone Posture Maneuver to Ameliorate Pusher Behavior in Acute Stroke: A Retrospective Study

**DOI:** 10.3390/jcm13247805

**Published:** 2024-12-20

**Authors:** Mitsuyo Ikeda, Syoichi Tashiro, Yusuke Harada, Kohei Ishita, Akifumi Masuda, Teruyuki Hirano, Shin Yamada

**Affiliations:** 1 Division of Rehabilitation Service, Kyorin University Hospital, Mitaka, Tokyo 181-8611, Japan; mtmori@ks.kyorin-u.ac.jp (M.I.); kouhei@ks.kyorin-u.ac.jp (K.I.); 2 Department of Rehabilitation Medicine, Kyorin University Faculty of Medicine, Mitaka, Tokyo 181-8611, Japan; a-masuda@ks.kyorin-u.ac.jp; 3 Department of Rehabilitation Medicine, Keio University School of Medicine, Shinjuku, Tokyo 160-8582, Japan; 4 Department of Rehabilitation, Faculty of Health Sciences, Kyorin University, Mitaka, Tokyo 181-8611, Japan; y-harada@ks.kyorin-u.ac.jp; 5 Department of Stroke and Cerebrovascular Medicine, Kyorin University, Mitaka, Tokyo 181-8611, Japan; terry@ks.kyorin-u.ac.jp

**Keywords:** attention, lateropulsion, exercise, postural disorder, stroke care unit, training

## Abstract

**Background:** Pusher behavior after stroke is an important sequela that interferes with rehabilitation and independence in activities of daily living. As represented by visual or vestibular feedback, conventional methods require substantial assistance and time commitments, but have limited effectiveness. A recent case series suggests that prone posture may alleviate pusher behavior in patients with acute stroke. This study was conducted to retrospectively investigate the effects of prone posture maneuvers. **Methods:** This retrospective cross-sectional observational study was conducted in a stroke care unit at a university hospital. In total, 37 acute stroke cases presenting with pusher behavior were included from 787 eligible patients. Individuals with pusher behavior were conditioned with prone posturing for 10 min for 2 consecutive days, in addition to regular daily rehabilitation training. The Scale for Contraversive Pushing (SCP) values, Stroke Impairment Assessment Set (SIAS), and functional activities were assessed before, immediately after, and three days after the intervention. **Results:** The SCP value and the ability to roll over and sit balanced significantly improved compared with the baseline (*p* < 0.05) and persisted for 3 days after the intervention. Multiple regression analysis identified the SIAS motor score as a determinant of SCP changes. **Conclusions:** The prone posture maneuver promptly and consistently suppressed pusher behavior, particularly in patients with mild paresis, as indicated by SCP values in acute stroke cases. The uncontrolled, single-site, and retrospective features of the current study require further investigation.

## 1. Introduction

Pusher behavior represents a significant postural localization disorder observed in approximately 10% of patients with stroke during the acute phase. The symptom is characterized by patients actively pushing away from their non-hemiparetic side, leading to loss of postural balance. This behavior results from the misperception of body orientation due to gravity, and the patients perceive their bodies as upright when they are tilted towards the side of the brain lesion. Pusher behavior is often underestimated due to its gradual improvement over time, and its features, mechanisms, and effective interventions still need to be studied [1,2]. Despite its gradual resolution, pusher behavior has notable implications, increasing the need for care and assistance during training, elevating the risk of falls, and impeding the transition from bed rest to active rehabilitation in the early phases [1,3]. Consequently, it is crucial to address pusher behavior during the early phase of stroke.

Several research groups have highlighted the impact of pusher behavior on stroke outcomes, revealing that it extends the time required to achieve specific levels of activities of daily living (ADL) compared with patients without pusher behavior [2,4]. Pederson et al. reported that, while pusher behavior did not directly influence functional outcomes, it doubled the rehabilitation period (3.6 weeks) needed to reach the same best ADL level as the control group [3]. Krewer et al. demonstrated that rehabilitation training for patients with pusher behavior was only half as efficient and effective as that for those without, resulting in a worse ADL prognosis [5]. In a study by Denells et al. involving 65 patients with hemiplegic stroke, 63% exhibited pusher behavior features at 1 week, with symptoms resolving at 6 weeks; however, 21% persisted for 3 months. Those with pusher behavior exhibited early severe neurological deficits, lower stroke severity scores, and significantly longer motor and functional recovery capacities than non-pushers [6]. Babyer et al. also noted that recovery from pusher behavior is prolonged when accompanied by proprioceptive, hemianopic visuospatial deficits, and motor deficits, as these deficits collectively impair postural control [4]. While the causative relationship between pusher behavior and prognosis can be challenging to discern, early symptomatic control is essential for effective rehabilitation and prevention of accidental injuries in patients with acute stroke.

Traditionally, compensatory approaches focusing on visual, vestibular, and proprioceptive inputs, which are often unaffected, have been applied to improve control of vertical upright orientation [1]. Although there have been a limited number of attempts to alternate these conventional treatments, many studies were conducted decades ago, and the majority of recent reports have been case series or reports. Consequently, the available treatment options for pusher behavior are limited in practice, with postural control training utilizing visual feedback, vestibular input, and somatosensory cues [1,7,8]. While some researchers have explored novel methods such as robot-assisted gait training [9] and machine-supported gait training [10], these approaches are not widely practical in routine clinical settings. Additionally, techniques involving visual feedback, vestibular input or somatosensory cues, while feasible, require therapist support and consume a significant portion of training time. Therefore, there is a need for new feasible approaches that can be easily integrated into general clinical practice without imposing a substantial burden on training time.

Recently, Fujino et al. reported that a prone posture maneuver for 10 min on 2 consecutive days effectively suppressed pusher behavior in three stroke patients during the early acute phase [11]. This maneuver presents itself as a promising treatment option for addressing pusher behavior, as it can be implemented at any medical institution without the need for special equipment, and does not require extensive support from therapists. This study retrospectively examined the outcomes of the prone posture maneuver and assessed its effectiveness in ameliorating pusher behavior in patients with acute stroke.

## 2. Materials and Methods

### 2.1. Study Approval and Inclusion Criteria

This retrospective cross-sectional observational study adhered to the principles outlined in the Declaration of Helsinki and was approved by the Ethics Committee of the University School of Medicine at our institute (Tokyo, Japan; No. 1469-01). To align with the ethical guidelines, an opt-out procedure was implemented. This study focused on patients who had experienced a stroke and were admitted to the stroke care unit at our institute (a university hospital) between October 2020 and January 2022. The prone posture maneuver was applied to patients meeting the following criteria: (1) exhibiting pusher behavior; (2) no longer confined to bed rest; (3) absence of musculoskeletal, respiratory, or circulatory disorders impeding the adoption of a prone posture; and (4) no severe cognitive impairment or neuropsychological symptoms. Notably, super acute treatments, such as recombinant tissue-type plasminogen activator (rt-PA), endovascular therapy, and surgery, were not considered within the inclusion and exclusion criteria.

### 2.2. Prone Posture Maneuver

**Figure 1:**
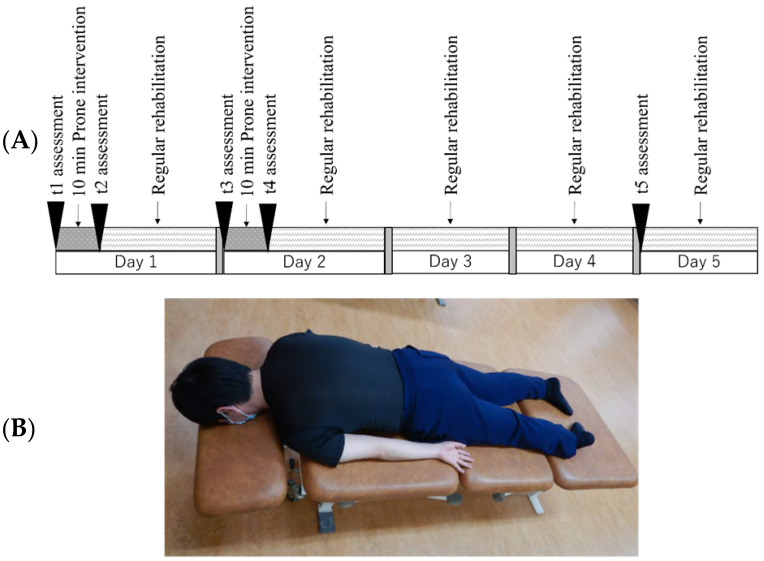
(**A**) Intervention timeline is shown. The prone posture maneuver was performed for two consecutive days. Clinical assessments, including the degree of pusher behavior, impairment, and functional ability, were performed before and after the intervention, and 3 days later. (**B**) A scene of the prone posture maneuver. On an open-faced bed, patients were placed in a prone posture for 10 min. An adjustable headrest is used to prevent neck rotation and extension. Initially, the therapist gently rocked the patient’s trunk and limbs to ensure relaxation. Patients were instructed to relax their necks and extremities as much as possible. A staff member reproduced this scene.

Upon the physician’s determination that patients were no longer restricted to bed rest, eligibility for intervention was assessed in accordance with the criteria established by the Japan Stroke Society board-certified attending physician. Patients meeting the eligibility criteria underwent the prone posture maneuver for two consecutive days, consistent with the approach used in the prior case series. The assessments were conducted before and after the 10 min prone posture maneuver and again 3 days post-intervention. The defined time points during the intervention were as follows: pre-intervention on the first day (t1, baseline), post-intervention on the first day (t2), pre-intervention on the second day (t3), post-intervention on the second day (t4), and three days after the intervention (t5) (Figure 1A). Notably, t1 served as the baseline, representing the period when the patients were unfamiliar with the prone posture maneuver.

For the prone posture maneuver, an open-faced bed SESAM VALLIANT (UA-14, OG Giken, Okayama, Japan) equipped with an adjustable headrest to prevent neck rotation and extension was used. The patients were asked to turn from the supine position to the non-paralyzed side. If there was strong resistance, a cushion was placed on the paralyzed side to encourage a gradual shift in the center of gravity to the non-paralyzed side. When the patient was induced to turn, the shoulder joint on the paralyzed side was protected to avoid placing inappropriate stress on the shoulder joint. Patients were positioned in a prone posture for 10 min with explicit instructions to relax their necks and extremities as much as possible (Figure 1B). At the commencement of the maneuver, the therapists gently facilitated trunk and limb rocking to promote relaxation. After the intervention, the patients underwent standard stroke rehabilitation as prescribed by the Japanese Association of Rehabilitation Medicine board-certified rehabilitation physicians. This rehabilitation regimen encompasses range of motion exercises, muscle strengthening training, functional training for paretic limbs, activities focusing on motor function (excluding prone posture), and activities targeting daily living functions. It is important to note that the period for the prone posture maneuver was considered part of the overall rehabilitation duration in this study, given the absence of a fixed definition for this maneuver.

### 2.3. Assessments

To ensure assessment validity, two therapists independently performed each evaluation, and in cases of score discrepancies, the lowest score was adopted.

(1) Pusher behavior

The Scale for Contraversive Pushing (SCP) was used to assess pusher behavior severity [12]. The SCP comprises three items (posture, extension, and resistance) scored on a scale of 0–1 based on severity, evaluated in sitting or standing postures, with a total score of 6 points. Patients with SCP scores > 0 in each section were considered to exhibit pusher behavior [13].

(2) Impairments

Neurological examinations for stroke impairments included the following assessments: Consciousness was assessed using the Glasgow Coma Scale (GCS). Motor impairments were evaluated using the Brunnstrom stage [14]. The motor and sensory functions and visuospatial perception were assessed using the Stroke Impairment Assessment Set (SIAS) [15,16]. Briefly, the SIAS motor items involved knee mouth, finger function, hip flexion, knee extension, and foot pat tests scored 0–5, reflecting motor performance due to paresis. The SIAS sensory items, including tactile sensation and positional function in the upper and lower extremities, scored 0–3. The tonus of the biceps, triceps, quadriceps, and biceps femoris muscles was evaluated bilaterally using the modified Ashworth Scale [17].

(3) Functional abilities

The motor functional activities were evaluated using six items of the Motor Assessment Scale (MAS), excluding the upper limbs and hands [18]. The MAS consists of supine-to-side lying (rolling), supine-to-sitting overside of bed (sitting up), balanced sitting, sitting-to-standing (standing up), and walking. Each item was rated on a scale of 0 to 6, and 0 was scored when the evaluation was inapplicable. The transfer ability was evaluated using the bed/wheel chair transfer items of the Japanese version of the Functional Independence Measure (FIM) version 3.0, which has culturally relevant modifications for some of the items [19,20].

### 2.4. Statistical Analysis

The sample size for this study was determined to detect differences in the superior colliculus point (SCP) change following the prone posture maneuver compared with the general recovery reported in previous studies that only applied conventional approaches to treat pusher behavior. The existing literature suggests a nearly linear reduction in SCP values between 10 and 45 days post-onset as part of natural recovery, with a mean value of 0.08–0.09 points/day [6]. Another study reported a SCP value decrease of 1.4 ± 1.0 points over three weeks in control patients undergoing regular rehabilitative training [21]. Considering an estimated natural recovery of 0.5 ± 0.5 points, a recovery after prone posture maneuver of 0.8 based on pilot trial findings, and setting alpha at 0.05 with a power of 95%, the calculated sample size was 36. The target recruitment range was set between 36 and 40 participants.

The Wilcoxon signed-rank test was employed to compare the immediate effect of a single session of the prone posture maneuver between t1 and t2, and the overall treatment effect of this intervention between t1 and t5 on functional activities and neurological examinations. The severity of pusher behavior throughout the intervention was assessed using the Friedman test for each SCP value at t1, t2, t3, t4, and t5, with multiple comparisons corrected using Bonferroni’s method. The relationship between SCP changes and neurological examination results was explored using Spearman’s rank correlation coefficient. Multiple regression analysis (stepwise method) was conducted with significantly correlated items as explanatory variables, and SCP change as the dependent variable. The significance level for each test was set at 5%, and SPSS Statistics Ver. 27 (IBM) was used for statistical analysis.

## 3. Results

### 3.1. Clinical Characteristics of Pusher Behavior in Acute Stroke

**Figure 2:**
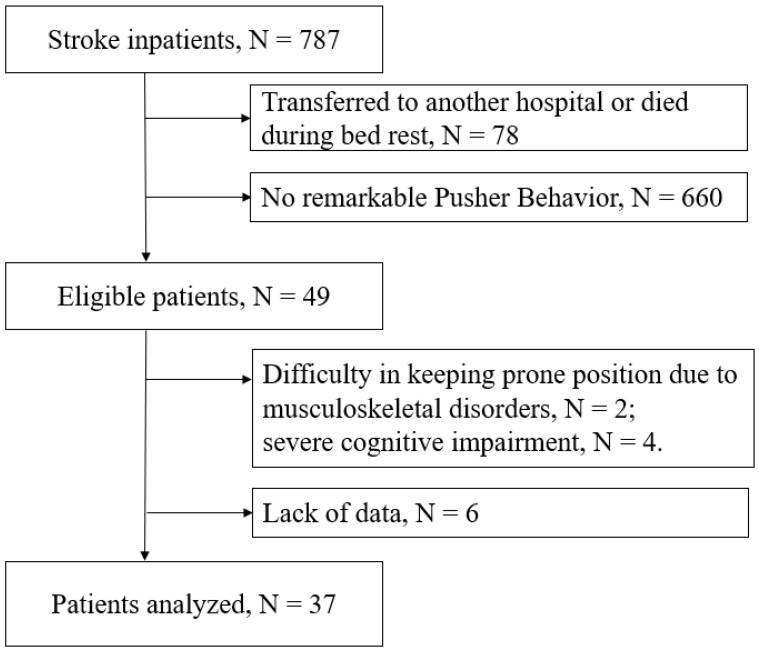
A flowchart showing patient selection. Among 787 consecutive patients with stroke, 49 who presented with pusher behavior were eligible for the intervention. After removing 6 individuals who fulfilled the exclusion criteria, 43 patients underwent conditioning with a prone posture maneuver. Since some of the clinical data were missing for 6 individuals, the data of the remaining 37 individuals were analyzed.

Of the 787 stroke patients treated in the hospital, 49 potential candidates were identified after excluding those who were transferred or deceased during the hyperacute phase and those without pusher symptoms. Six patients were deemed ineligible due to musculocutaneous disorders or severe cognitive problems. After addressing data-related issues for 6 patients, a total of 37 individuals (22 men and 15 women) were included in the retrospective study (Figure 2). No dropouts occurred, and the patient demographics are presented in Table 1.

### 3.2. Changes in Pusher Behavior, Impairments, and Functional Activities

**Figure 3:**
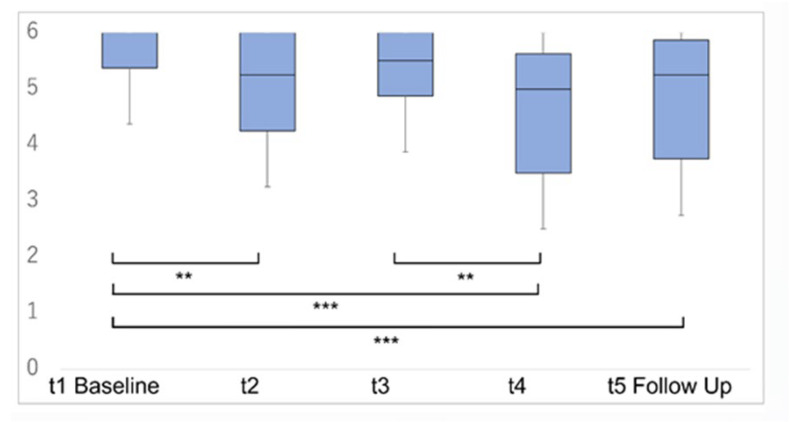
Time transition of SCP scoring. An immediate significant improvement in SCP score was observed after prone posture maneuver each day. A significant improvement compared with the baseline was also observed in the follow-up assessment at t5. *** Bonferroni corrected *p* < 0.001. ** Bonferroni corrected *p* < 0.01.

SCP values exhibited significant improvement over the entire observation period, including the immediate effect of the prone posture maneuver and the lasting effect for three days (Figure 3). Immediate effects, assessed within the 10 min interval between t1 and t2, revealed significant improvements in SCP values and motor functional abilities related to rolling and balanced sitting in MAS (*p* < 0.05). No significant differences were observed in GCS, motor palsy, sensory function, hemispatial neglect, muscle tonus of paretic limbs, and functional abilities related to sitting up, standing up, and walking in MAS, and the FIM transfer score (Table 2, middle column).

Lasting effects for 3 days after the interventions, assessed between t1 and t5, showed significant improvements (*p* < 0.05) in SCP values, upper limb tactile perception, all items in MAS basic movements, and the FIM transfer score. However, lasting effects may include natural recovery. Conversely, no differences were noted in GCS score, motor palsy, hemispatial neglect, and muscle tonus (Table 2, right column).

### 3.3. Relationship Between Changes in Pusher Behavior and Impairments

To characterize the impairment of patients for whom the prone posture maneuver would be effective, the relationship between background status and SCP changes was examined. The immediate SCP change between t1 and t2 was significantly correlated with the baseline SCP, SIAS-m score, and superficial sensation at t1. Multiple regression analysis with these factors revealed that SIAS-m (β = −0.49) was a significant determinant. The analysis showed a Durbin–Watson ratio of 1.48, VIF of 1.0, and adjusted R2 of 0.22.

In contrast, the lasting SCP change between t1 and t5 was significantly correlated with SIAS-m, superficial sensation, positional, GCS, and MAS scores for sitting up and walking. Multiple regression analysis using these factors identified SIAS-m (β = −0.60) as the determinant, with a Durbin–Watson ratio of 1.89, VIF of 1.0, and adjusted R2 of 0.34.

## 4. Discussion

The findings of this study underscore the immediate and lasting effectiveness of short and less frequent prone posture maneuvers in mitigating pusher behavior, as evaluated through SCP values. The improvements observed in SCP values, MAS rolling, and MAS balanced sitting, and the decrease in muscle tonus on the nonparetic side immediately after the initial intervention, highlight the impact of the maneuver on relevant aspects of postural control and functional activities. Notably, this study distinguishes between the immediate effect, observed between t1 and t2, and the lasting effect, spanning from t1 to t5. While the immediate effect primarily captures the direct influence of the prone posture maneuver, the lasting effect may incorporate the natural recovery over time. The rate of SCP improvement in this study (0.2/day), exceeding that reported in a prior investigation of acute stroke patients undergoing conventional rehabilitation (0.08–9) [6], suggests the potential of the prone posture maneuver to rapidly alleviate pusher behavior. This, in turn, supports its role in preparing patients with pusher behavior for rehabilitation and expediting the transition to early bed leaving and rehabilitation.

Pusher behavior remains a significant impediment to the recovery process in acute stroke [4], and evidence-based interventions are limited. Pusher behavior is commonly linked to sensory perception dysfunction and impaired vertical postural control [21]. Karnath et al. reported that primary neural disruption occurs in the posterolateral thalamus in patients presenting with Pusher behavior, which causes patients to perceive their body as upright when it is tilted towards the side of the brain lesion. Conventional rehabilitation maneuver facilitates the patients to process verticality correctly with preserved sensory modalities like visual, vestibular, and proprioceptive inputs [1]. It is recognized that more than visual verticality feedback is required. A recent clinical practice recommendation by a Delphi expert panel proposes more concrete features of specific rehabilitation strategies, including usage of cues, treatments to address neglect, a combinatorial approach with visual feedback using mirror or alignment and proprioceptive and graviceptive input or tactile and verbal reference, and high-level systemic motor execution like standing or walking [22]. Furthermore, recent studies have explored alternative treatments to suppress pusher behavior, including galvanic vestibular stimulation, machine-supported gait training [10], and computer-assisted interactive visual feedback training [23]. Despite promising results, these new strategies come with challenges related to cost and preparation time. Another group investigated the effect of inhibitory repetitive transcranial magnetic stimulation (rTMS) on the intact inferior parietal lobe, but no remarkable effect on pusher behavior was observed [24]. On the other hand, the Delphi panel recommendation further suggests favorable “general care” like activities to encourage weight shift onto and movement directed to the less affected side to address the fear of falling, consistent cueing by caregivers, external reference of true vertical, positioning upright as soon as possible, usage of a well-fitted wheelchair, and to provide the frequent change of position throughout the day [22].

Fujino et al. hypothesized that the extended supine posture disrupts muscle tonus equilibrium, reinstates extensor muscle activity, and diminishes excessive motor output in the nonparetic upper and lower limbs and trunk [11]. It is not rare that the acute stroke patients spend extended periods in bed due to dismobility and intensive treatment, which might lead to disruption of the muscle tonus balance between extensors and flexors, and this deviation induces a disuse of vertical perception. Interestingly, a degree of reduction in muscle tone in the nonparetic limb was observed in the current study, with some patients experiencing a significant suppression of trunk extensor muscle tonus, resulting in forward collapse after sitting up from the supine posture. In contrast, other clinical parameters, such as paresis, tactile sensation, proprioception, and tonus on the paretic side, did not show significant changes following the prone posture maneuver. This might explain why patients with mild motor paresis without flaccid paralysis gained greater benefit from the prone posture maneuver. Furthermore, the hemispatial neglect is sometimes considered a cause of pusher behavior [3,25], although there is controversy [1]. The current study does not find a significant effect on hemispatial neglect scoring, in line with Baier et al.’s findings [26]. This suggests that the beneficial effect of the prone posture maneuver may be independent of the recovery of hemispatial neglect.

Although the prone posture maneuver is currently not widely recognized or included in major textbooks, consensus papers, or recent reviews of new approaches [22,27], it is noteworthy that the principle may be common to a part of the Delphi panel recommendations, i.e., to promote positioning in a well-fitted wheelchair and facilitate a change in position throughout the day [22]. The results of this study emphasize the maneuver’s potential as a standard method to mitigate pushing behavior before rehabilitative training, encompassing facilitation, functional activities, and ADL. In addition, it is implicated that the prone posture maneuver and conventional methods, including approaches targeting hemispatial neglect, will act collaboratively upon the difference in their mechanisms. Further research and recognition within the rehabilitation community may help integrate this maneuver into standard practice, contributing to improved outcomes for patients of stroke presenting with pusher behavior.

### Limitations

This study had several limitations that warrant consideration. First, it was a single-center observational study without a control group. Although the effect of the prone posture maneuver seemed greater than that of machine rehabilitation [10], it was generally difficult to compare the current results with those of other studies because there have been only a small number of qualified studies on pusher behavior. Most of the recent reports are case series, and even in the case of research, they are generally old and do not seem sufficiently qualified. While a randomized controlled trial will provide more robust evidence on the effectiveness of the maneuver, an N of 1 approach, where the patient becomes his/her own control, may be more suited to investigate pusher behavior because of its complex features and individual differences. In addition, generalizability to other countries, facilities, and patient groups might also be limited. For example, our target population included a relatively small fraction of patients presenting with pusher behavior (6.2%). We consider this to be because our facility accepts all stroke types, including asymptomatic TIA, in a country where a universal medical insurance system is established. In fact, a cross-sectional survey from our country reported a similar rate (8.0%) [28]. We believe that our criteria to diagnose pusher behavior are common and will ensure the severity of the symptoms and the solid characteristics of the patients; however, the difference in patient population may hinder the generalizability of the results. Second, the optimal protocol for prone posture treatment remains unclear. Whereas this study traced the protocol of a prior case series [11], investigations into the longer-term effects and the benefit of implementing a longer duration or more frequent maneuvers are needed to enhance the maneuver’s applicability. This limitation may be rooted in the features of the current intervention, which was implemented by rehabilitation therapists. Although we did not count this intervention as part of rehabilitation training, the time of therapists is generally costly in a strict manner. Therefore, we would expect further research to optimize this maneuver by nurses as a part of nursing care. Third, the small sample size did not allow us to implement subgroup analysis in relation to the lesion and its laterality of brain damage. This limitation impedes seeking the condition to be a responder or the mechanism of the current approach. Finally, the study does not thoroughly elucidate the mechanisms of the prone posture maneuver, with a suggestive implication that it suppresses pusher behavior by modulating muscle tonus on the nonparetic side and trunk. Assessing back muscle tonus through myotonometry or neurophysiological testing, together with an assessment of subjective visual vertical and subjective postural vertical using a tilting chair [12,29], may provide valuable insights. Furthermore, the means to evaluate higher level cognitive function from various aspects, including the Catherine Bergego scale [30] for hemispatial neglect, will also be required.

## 5. Conclusions

This uncontrolled single-center observational study in a stroke unit showed that a short conditioning with a prone posture maneuver effectively suppressed pusher behavior as assessed by SCP values in patients with acute stroke. Our regimen for two consecutive days induced not only an immediate but also a lasting effect for a week. Patients with milder paresis tended to become good responders. It was implied that the amelioration of trunk muscle tone was one of the mechanisms of pusher behavior suppression. The prone posture maneuver has the potential to be a good clinical approach for pusher behavior, together with conventional approaches utilizing visual and vestibular inputs.

## Figures and Tables

**Table 1:** Participant demographics (n = 37).

	N(SD), or Median (IQR)
Age (years)	72.7 (13.4)
Sex (male/female)	22/15
Type of stroke (hemorrhage/ischemia/other)	23/10/4
Affected hemisphere (right/left)	23/14
Intervention date from the onset	13.0 (9.0–20.0)
Length of hospital stay	44.9 (17.6)
NIHSS at admission	18.4 (9.6)

IQR—interquartile range; NIHSS—National Institutes of Health Stroke Scale; SD—standard deviation.

**Table 2:** Immediate and lasting changes in impairments and functional abilities over the intervention period.

	t1	t2	*p* (t1–t2)	t5	*p* (t1–t5)
GCS	13 (11–14)	14 (11–14)	0.08	14 (12–14)	0.08
Brunnstrom stage (total)	6 (6–7)	6 (6–7)	1.0	6 (6–7)	1.0
SIAS					
Motor items (total)	0 (0–7)	0 (0–7)	1.0	0 (0–7)	1.0
Sensory items superficial U/E	0 (0–1)	0 (0–1.25)	1.0	0 (0–2)	0.03 *
Superficial L/E	0 (0–1)	0 (0–1.25)	0.32	0 (0–1)	0.32
Position U/E	0 (0–1)	0 (0–0.5)	1.0	0 (0–1)	0.32
Position L/E	0 (0–1)	0 (0–1)	0.56	0 (0–1)	0.71
Visuospatial	2 (1–2)	1 (0.5–2)	0.16	1 (1–2)	1.0
Muscle Tone (mAS)					
Elbow flexors	1 (0–2.5)	1 (0–1)	0.08	1 (0–1)	0.10
Elbow extensors	0 (0–1)	0 (0–1)	0.66	0 (0–1)	0.15
Knee flexors	1 (0–1)	1 (0–1)	0.32	1 (0–1)	1.0
Knee extensors	0 (0–0)	0 (0–1)	1.0	0 (0–0)	0.16
Elbow flexor (nonparetic)	1 (0–1.5)	1 (0–1)	0.09	1 (0–1)	0.14
Elbow extensor (nonparetic)	1 (0–1)	0 (0–1)	0.01 *	1 (0–1)	0.42
Knee flexor (nonparetic)	1 (1–1)	1 (0–1)	1.0	1 (0–1)	1.0
Knee extensor (nonparetic)	1 (0–1)	0 (0–0)	0.01 *	1 (0–1)	1.0
MAS					
Rolling	0 (0–0)	0 (0–1)	0.02 *	0 (0–1)	0.01 *
Sitting up	0 (0–1)	0 (0–1)	0.11	0 (0–1)	0.01 *
Balanced sitting	1 (1–2)	2 (1–2)	0.00 *	2 (1–2)	0.00 *
Standing up	0 (0–1)	0 (0–1)	0.06	1 (0–1)	0.00 *
Walking	0 (0–0)	0 (0–0)	0.32	0 (0–0)	0.02 *
FIM transfer	1 (1–1)	1 (1–1.5)	0.32	1 (1–2.5)	0.04 *

Note: (n = 37). * Wilcoxon’s signed rank test (values < 0.05). Median (interquartile range). FIM—Functional Independence Measure, GCS—Glasgow Comma Scale, MAS—Motor Assessment Scale, mAS—modified Ashworth Scale, SIAS—Stroke Impairment Assessment Set, t1: baseline, t2: immediately after the first intervention, t5: three days after the initial intervention.

## Data Availability

Because of the ethical approval for this retrospective study, we cannot share the raw experimental data unless by obtaining written consent from each participant.
